# AZD0530 sensitizes drug‐resistant ALK‐positive lung cancer cells by inhibiting SRC signaling

**DOI:** 10.1002/2211-5463.12162

**Published:** 2017-03-10

**Authors:** Yi Zhao, Yi Yang, Yunhua Xu, Shun Lu, Hong Jian

**Affiliations:** ^1^ Shanghai Lung Tumor Clinical Medical CenterShanghai Chest HospitalShanghai Jiao Tong UniversityChina

**Keywords:** ALK, AZD0530, NSCLC, resistance, SRC

## Abstract

Most tumors develop resistance to targeted cancer drugs, even though these drugs have produced substantial clinical responses. Here we established anaplastic lymphoma kinase (ALK)‐positive drug‐resistant lung cancer cell lines, which are resistant to ceritinib (LDK378). We found that ceritinib treatment resulted in robust upregulation of SRC activity, as measured by the phosphorylation of the SRC substrate paxillin. Knockdown of SRC alone with siRNA effectively sensitized ceritinib resistance in ALK‐positive cells. Furthermore, SRC inhibition by AZD0530 was effective in ALK‐resistant cancer cells. Thus, ALK inhibition by ceritinib may lead to upregulation of SRC signaling, and AZD0530 could serve as a potential drug in the clinic to treat ALK‐resistant lung cancer patients.

**Abbreviations**
ALK: anaplastic lymphoma kinase
FBS: fetal bovine serum
NSCLC: non‐small cell lung cancer
PBS: phosphate‐buffered saline
RPMI1640: Roswell Park Memorial Institute 1640 medium
SRC: proto‐oncogene SRC, Rous sarcoma
TKIs: tyrosine kinase inhibitors

Lung cancer causes the most cancer deaths world‐wide 1. Approximately 85% of lung cancer is non‐small cell lung cancer (NSCLC) 2. Current chemotherapeutics for NSCLC have very low efficacy 3, 4.

Genotype‐based targeted therapies have had a significant success in the treatment of cancers 5, 6. Anaplastic lymphoma kinase (ALK) rearrangement is a therapeutically tractable oncogenic driver that occurs in 2–7% of NSCLC patients 7, 8. So far, several ALK‐targeted small‐molecule tyrosine kinase inhibitors (TKIs) have been approved and successfully inhibit tumor growth 9, 10. However, usually within 1–2 years, cancer cells eventually develop resistance to these inhibitors through a variety of mechanisms 11, 12.

Resistant cells can develop secondary resistance mutations in ALK preventing target inhibition by the corresponding TKI 13, 14 or a compensatory signaling pathway that re‐establishes activation of key downstream proliferation and survival signals 15, 16. As more drugs are developed that effectively overcome secondary resistance mutations in the targeted genes, these bypass track mechanisms of resistance will likely continue to emerge in clinical practice.

Signaling by SRC (a proto‐oncogene tyrosine‐protein kinase encoded by the *SRC* gene, similar to the v‐*Src* gene of Rous sarcoma virus) is known to be a focal point of integrin‐mediated signaling and the transduction of extracellular signals and plays a key role in tumorigenesis and metastatic progression 17. Over‐expressed and/or hyper‐activated SRC is caused by enhanced expression or dysregulation of upstream growth factor receptors and non‐receptor tyrosine kinases 18. However, whether SRC functions in drug‐resistant ALK‐positive NSCLC and could be a potential target is still unknown.

In our current study, we established ceritinib‐resistant NSCLC cells *in vitro* and further studies suggested that SRC plays a critical role in cells with ceritinib‐resistance. We examined that inhibition of SRC signaling by AZD0530 (SRC inhibitor) could block cell proliferation to overcome ceritinib resistance in ALK‐positive NSCLC cells.

## Materials and methods

### Cell culture

Human NSCLC cell lines H3122 and H2228 (ATCC, Manassas, VA, USA), were grown in RPMI1640 medium (Invitrogen, Carlsbad, CA, USA) supplemented with 10% fetal bovine serum (FBS; Gibco, Carlsbad, CA, USA). Cells were maintained at 37 °C under 5% CO_2_.

### Establishment of ceritinib‐resistant ALK‐positive cell lines

Ceritinib‐resistant cells were developed by chronic, repeated exposure of H3122 and H2228 cells to ceritinib. Over a period of 6 months, H3122 and H2228 cells were continuously exposed to ceritinib. The concentration of ceritinib was increased twofold every month until it reached 100 nm. The medium containing ceritinib was changed every 3 days. Cells were passaged when the surviving cells grew to 90% confluence. The cells were thereafter maintained in 100 nm ceritinib.

### RNAi and transient transfection

The SRC RNAi sequence was: 5′‐AAG TGC GGC CAT TTC ACC AGC‐3′. The AKT RNAi sequence was: 5′‐AAC CTC ACT ATG GTA TGC TGG‐3′. The scramble sequence was: 5′‐CGA GTT GTA GAT CCT CAT A‐3′. For transfection, the day before transfection, cells were seeded in six‐well plates and grown overnight when the cells had reached 75% confluence. Transient transfections were performed with scramble siRNA oligomers or siRNA oligomers targeting SRC mRNA using RNAiMAX (Invitrogen) according to the manufacturer's protocol.

### Western blotting

Cells were lysed in RIPA buffer containing proteinase inhibitor cocktail and phosphatase inhibitor cocktail (Sigma‐Aldrich, St. Louis, MO, USA); after clarification by centrifugation at 12 000 ***g***, 30 μg samples were loaded into each lane of 4–12% polyacrylamide gels. Antibodies against‐ALK, phosphor‐ALK (Y1604), AKT, phosphor‐AKT (S473), SRC, phosphor‐SRC (Y416), Paxillin, phosphor‐paxillin (Y118) and β‐actin (all from Cell Signaling, Beverly, MA, USA) were used and detected by the chemiluminescence reaction carried using a Pierce ECL kit (Pierce, Rockford, IL, USA).

### Cell proliferation assay

Cells were seeded in 24‐well plates at low density (1 × 10^4^ per well), and cultured overnight. Then cells were treated with the indicated drugs and cultured for 4 days. Then 20 µL 3‐(4,5‐dimethylthiazol‐2‐yl)‐2,5‐diphenyl‐tetrazolium bromide (MTT) (5 mg·mL^−1^) (Sigma‐Aldrich) was added into each well, and the cells were incubated for a further 4 h. The absorbance was recorded at A570 nm with a 96‐well plate reader after DMSO addition.

### Statistical analysis

Data are the mean ± SD of at least three independent experiments. All data were analyzed with spss statistics version 15 (SPSS Inc., Chicago, IL, USA). Independent two‐group analyses were performed with Student's *t*‐test. Differences were considered significant when *P* < 0.05.

## Results

### Ceritinib‐resistant NSCLC cells show increased cell growth

**Figure 1:**
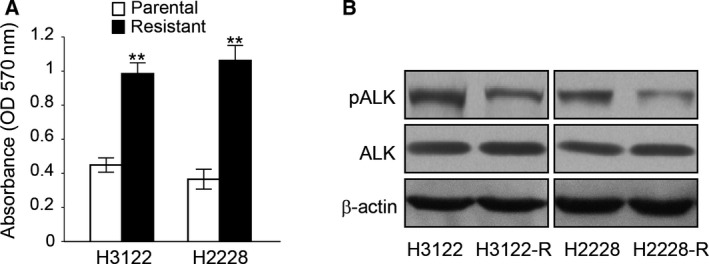
Ceritinib‐resistant NSCLC cells show increased cell growth. (A) MTT assay showing cell growth of H3122 and H2228 parental and ceritinib‐resistant cells. **P < 0.01. (B) Western blot for phosphor‐ALK and total ALK in H3122 and H2228 parental and ceritinib‐resistant cells.

The ceritinib‐resistant NSCLC cell lines (H3122‐R and H2228‐R) were established by long‐term ceritinib treatment. We characterized the ceritinib‐resistant cells by examining cell proliferation and cell invasion capability. MTT assay results showed that ceritinib treatment significantly promoted the proliferation of H3122‐R/H2228‐R cells compared with parental H3122/H2228 cells (Fig. 1A). Western blotting showed that in H3122‐R/H2228‐R cells, phosphorylation of ALK is decreased compared with cells without ceritinib treatment (Fig. 1B). These results indicated that continuous ceritinib treatment could inhibit phosphorylation of ALK and lead to activation of other pathways to promote NSCLC cell growth.

### SRC signaling pathway is activated in ceritinib‐resistant NSCLC cells

**Figure 2:**
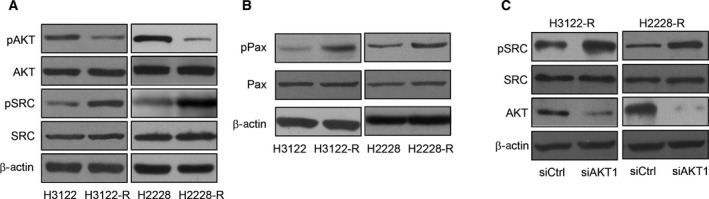
SRC signaling pathway is activated in ceritinib‐resistant NSCLC cells. (A) Western blot for phosphor‐AKT, total AKT, phosphor‐SRC and total SRC in H3122 and H2228 parental and ceritinib‐resistant cells. (B) Western blot for phosphor‐paxillin and total paxillin in H3122 and H2228 parental and ceritinib‐resistant cells. (C) Western blot for phosphor‐SRC and total SRC in AKT knockdown H3122 and H2228 ceritinib‐resistant cells compared with control cells.

Abnormal activation of the phosphoinositide 3‐kinase (PI3K)/protein kinase B (AKT) pathway is one of the most common tumor‐related signaling abnormalities that regulates cell proliferation and invasive capability, and it can be detected in a variety of tumors. Western blotting showed in H3122‐R/H2228‐R cells, ALK inhibition primarily impacted PI3K signaling, which resulted in upregulation of SRC signaling (Fig. 2A). As SRC signaling is known to be a focal point of integrin‐mediated signaling and the transduction of extracellular signals, we determined that robust upregulation of SRC activity by ALK inhibition induced high phosphorylation of paxillin in ceritinib‐resistant cells (Fig. 2B). Furthermore, SRC signaling was also upregulated by inhibition of phosphorylation of AKT, which is downstream signaling pathway of ALK (Fig. 2C). Overall these results demonstrated that the SRC signaling pathway is highly activated in ceritinib‐resistant NSCLC cells.

### SRC mediates resistance in ALK‐positive NSCLC cells

**Figure 3:**
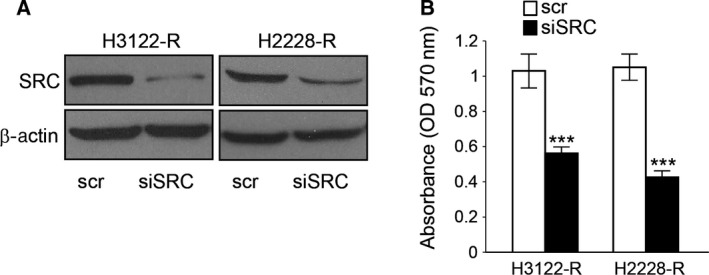
SRC mediates resistance in ALK‐positive NSCLC cells. (A) Western blot showing SRC knockdown efficiency in ceritinib‐resistant H3122 and H2228 cells. Scr: scramble siRNA. (B) MTT assay showing cell growth in SRC knockdown H3122 and H2228 ceritinib‐resistant cells compared with control cells. ***P < 0.001.

To determine whether SRC plays a role in ceritinib resistance, we performed SRC knockdown with siRNA in H3122‐R/H2228‐R cells (Fig. 3A). MTT assay results (Fig. 3B) showed that cells with SRC inhibition were sensitized to ALK inhibition, demonstrating that SRC inhibition is sufficient to re‐sensitize cells to ALK inhibition. These results confirmed that ceritinib activated the SRC signaling pathway, and the SRC signaling pathway is necessary for ceritinib resistance in ALK‐positive NSCLC cells.

### AZD0530 inhibits proliferation in ceritinib‐resistant ALK‐positive NSCLC by blocking SRC activity

**Figure 4:**
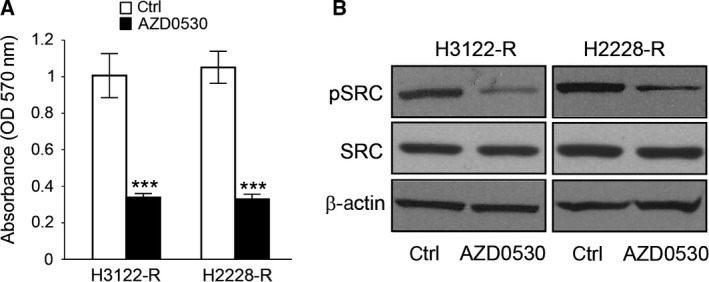
AZD0530 inhibits proliferation in ceritinib‐resistant ALK‐positive NSCLC cells by blocking SRC activity. (A) MTT assay showing cell growth in AZD0530 treated H3122 and H2228 ceritinib‐resistant cells compared with control cells. ***P < 0.001. (B) Western blot for phosphor‐SRC and total SRC in AZD0530 treated H3122 and H2228 ceritinib‐resistant cells compared with control cells.

Next we examined the effect of combined ALK and SRC inhibition on resistant ALK‐positive cells. All the groups were cultured in media with ceritinib to maintain ALK resistance selectivity. Then cells in the experimental group were treated with AZD0530 (an inhibitor of SRC) while cells treated with PBS served as a negative control. MTT assay showed these resistant ALK‐positive cell lines were highly sensitive to AZD0530 in the presence of ceritinib, which resulted in loss of cell growth (Fig. 4A). Consistent with these results, ceritinib failed to inhibit downstream SRC signaling except in the presence of AZD0530 in these resistant cells (Fig. 4B). These results demonstrate that AZD0530 treatment could inhibit proliferation in ceritinib‐resistant ALK‐positive NSCLC cells by inhibiting SRC activity.

## Discussion

In this study, we established ceritinib‐resistant ALK‐positive cell lines *in vitro*. Although ceritinib strongly inhibited the phosphorylation of ALK (Fig. 1B) in H3122 and H2228 parental cells as well as H3122‐R and H2228‐R cells, the resistant cells acquired higher proliferation capability than parental cells (Fig. 1A).

H3122 cells resistant to ceritinib via epithelial‐mesenchymal transition were demonstrated previously 19. In that study, epithelial markers decreased while the expression of Vimentin, Snail, Notch, Caveolin, and SRC were upregulated. Consistent with these data, our results showed that the level of phosphorylation of SRC was highly upregulated in ceritinib‐resistant H3122‐R and H2228‐R cells (Fig. 2A). To show SRC signaling is activated, we found phosphorylation of paxillin is upregulated (Fig. 2B) suggesting tumor progression. We also demonstrated that AKT is the downstream regulator of ALK signaling which regulates SRC activity (Fig. 2C). Most importantly, we demonstrated that SRC knockdown sensitizes H3122‐R and H2228‐R cells to ceritinib treatment (Fig. 3).

AZD0530, an SRC inhibitor, has been reported to exhibit clinical activity in cancer patients 20. Our results showed that both H3122‐R and H2228‐R are sensitive to AZD0530 in the *in vitro* cell proliferation assay, compared with the control group (Fig. 4).

In conclusion, we clearly demonstrated that SRC activity is upregulated and plays critical role in promoting cell growth in ceritinib‐resistant ALK‐positive NSCLC cells. Further, AZD0530 was efficacious against SRC activation. It is expected that AZD0530 will be a potent therapeutic option for patients with ALK‐TKI‐resistant tumors.

## Author contributions

YZ and YY conducted the experiment and drafted the manuscript. YX analyzed the data and participated in the experiments. SL and HJ conceived the project and guided the project's progress.
